# Facile Synthesis of Biomass N, S-CDs for Fluorescent Detection of Tetracycline in Wastewater

**DOI:** 10.3390/molecules31122014

**Published:** 2026-06-09

**Authors:** Bo Yu, Suchang Zou, Tianle Wang, Feng Guo, Weilong Shi, Zhimin Ao

**Affiliations:** 1 Guangdong Provincial Key Laboratory of Wastewater Information Analysis and Early Warning, Beijing Normal University, Zhuhai 519087, China; 2 School of Material Science and Engineering, Jiangsu University of Science and Technology, Zhenjiang 212003, China; 3 School of Energy and Power, Jiangsu University of Science and Technology, Zhenjiang 212003, China

**Keywords:** biomass, shuttlecocks, N, S-CDs, tetracycline, fluorescence detection

## Abstract

As the growing presence of antibiotic residues in environmental water bodies poses an increasing risk to ecological safety and human health, developing simple and efficient methods for the targeted detection of antibiotics is of particular importance. In this study, we propose a simple method for the one-step hydrothermal synthesis of N, S-co-doped carbon dots (N, S-CDs) using disulfide bonds from discarded badminton shuttlecocks. We investigated the effects of different synthesis temperatures on its performance and confirmed the method’s excellent performance in detecting tetracycline (TC) concentrations, with results demonstrating that varying synthesis temperatures affect the degree and distribution of carbonization, thereby influencing fluorescence intensity. Consequently, employing N, S-CDs-180, which exhibits optimal photoluminescence properties, as the sensing probe for the detection of TC solutions at varying concentrations yielded an excellent linear equation for fluorescence quenching and the detection limit is 1.963 mg/L. Additionally, the fluorescence stability of N,S-CDs-180 was investigated in laboratory water, tap water, seawater, lake water, and industrial wastewater, all of which demonstrated exceptional environmental adaptability. Furthermore, a systematic investigation into the target selectivity of N, S-CDs-180 toward various antibiotics revealed that this material exhibits a sensitive quenching response specifically to tetracycline-class antibiotics while showing no quenching effect on non-tetracycline antibiotics, collectively indicating that the as-prepared N, S-CDs can serve as potential fluorescent probes for the highly selective detection of tetracycline-class antibiotics in complex aqueous systems.

## 1. Introduction

With the widespread use of antibiotic pharmaceuticals, substantial quantities of antibiotics have entered aquatic environments. Over time, this poses inevitable risks to both natural ecosystems and public health [1]. Tetracycline (TC), owing to its bacteriostatic activity, is frequently employed to treat a variety of bacterial infections—particularly atypical pathogen infections that are unresponsive to other antibiotics—and is extensively used in aquaculture and livestock farming [2]. Because of its large-scale and often irrational use, considerable amounts of tetracycline residues persist in water bodies, far exceeding established national safety thresholds and posing hazards to human health [3]. Therefore, the development of rapid, stable, and sensitive methods for the monitoring and regulation of tetracycline is of critical importance.

To date, a variety of methods have been employed for the quantitative detection of tetracycline, including liquid chromatography–tandem mass spectrometry (LC-MS/MS) [4], high-performance liquid chromatography (HPLC) [5], enzyme-linked immunosorbent assay (ELISA) [6], and spectrophotometry [7]. Although each of these techniques possesses distinct advantages, they also suffer from limitations such as high instrumental costs, complex operational procedures, and insufficient sensitivity, which collectively hinder the widespread implementation of TC detection. Consequently, there is an urgent need to develop a low-cost and highly sensitive method for TC detection.

Fluorescent probes have garnered considerable attention owing to their low cost, high sensitivity, and favorable visualization capability [8]. However, single-emission fluorescent probes generally exhibit limited selectivity and are susceptible to interference from other factors [9]. To improve their performance, doped fluorescent probes have been increasingly investigated [10]. The core advantage of such probes lies in the simultaneous incorporation of two or more elements, which allows the modulation of the electronic structure, active sites, and the number of surface functional groups through synergistic effects, thereby enhancing their fluorescence properties [11]. Moreover, the emission spectrum of dual-element-doped probes can overlap more extensively with the absorption spectrum of TC, leading to a higher energy transfer efficiency from the probe to TC and, consequently, substantially improved selectivity.

In this study, a nitrogen and sulfur dual-doped carbon dot (N, S-CDs) fluorescent probe was designed for the simple and efficient detection of TC in wastewater. The probe was synthesized from commonly available badminton shuttlecocks via a one-step hydrothermal method, and the fluorescence on–off quenching response was achieved through the inner filter effect of TC on the excitation light. Within a certain concentration range, the concentration of TC can be quantitatively described by a linear equation relating to the fluorescence quenching efficiency. This work therefore demonstrates a green, cost-effective strategy for converting discarded sports equipment into functional fluorescent probes, offering a promising platform for the rapid, selective, and on-site monitoring of tetracycline residues in complex water environments.

## 2. Results and Discussion

**Figure 1:**
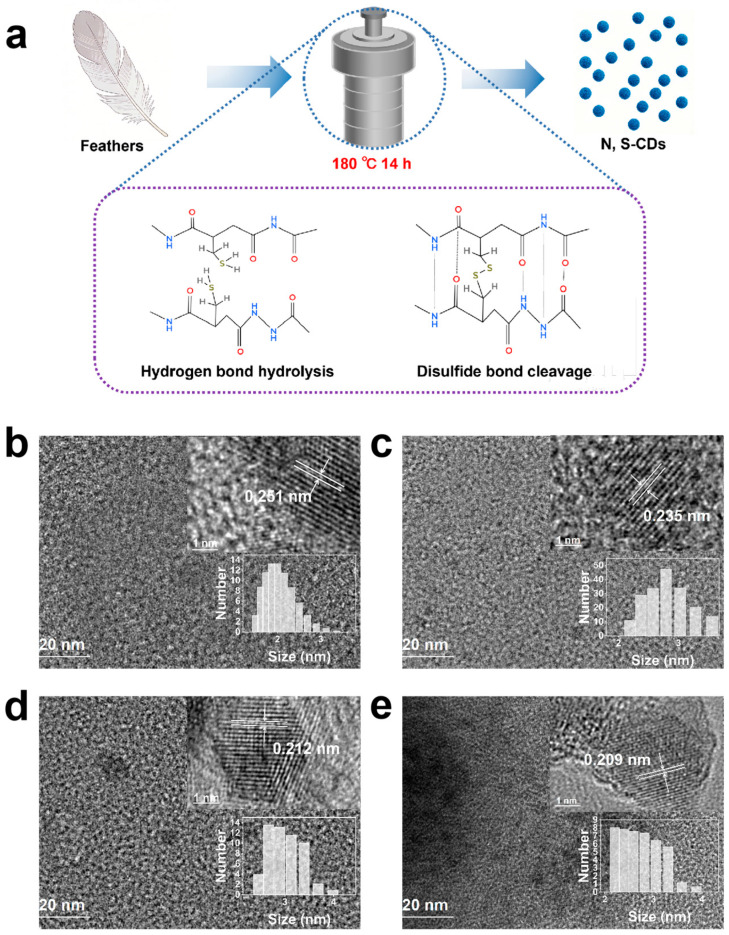
(**a**) Schematic diagram of the synthesis steps for N, S-CDs. TEM, HRTEM images and corresponding particle size distribution of (**b**) N, S-CDs-160, (**c**) N, S-CDs-180, (**d**) N, S-CDs-200 and (**e**) N, S-CDs-220.

In this study, nitrogen and sulfur co-doped carbon dots (N, S-CDs) were synthesized from feathers via a hydrothermal method [12], as illustrated in Figure 1a. Under high-temperature (160–220 °C) and high-pressure conditions, keratin macromolecules in feathers undergo hydrolysis, yielding small molecular species such as amino acids, accompanied by the cleavage of disulfide bonds [13]. These intermediates subsequently undergo dehydration and condensation reactions, gradually forming polymeric precursors enriched with nitrogen and sulfur, thereby enabling in situ heteroatom doping. As the reaction progresses, the precursors undergo further carbonization. During this process, nitrogen atoms are predominantly incorporated in the form of pyridinic nitrogen, while sulfur atoms are integrated into the carbon framework as thiophene-like or pyrrole-like sulfur species [14]. Ultimately, structurally stable N, S-CDs with uniformly distributed nitrogen and sulfur elements are obtained. Transmission electron microscopy (TEM) was employed to investigate the morphology of the N, S-CDs synthesized at different hydrothermal temperatures. As shown in Figure 1b–e, the N, S-CDs prepared at all four temperatures exhibited spherical morphologies and consistent lattice spacings of approximately 0.21 nm, corresponding to the (100) plane of graphitic carbon [15], indicating the high crystallinity of the as-prepared N, S-CDs. Further statistical analysis of particle size revealed that the particle size distributions of N, S-CDs-160, N, S-CDs-180, N, S-CDs-200 and N, S-CDs-220 ranged from 1.3 to 3.3 nm, 2 to 4 nm, 2 to 4 nm, and 2.2 to 4 nm, respectively, with corresponding average particle sizes of 2.06 ± 0.25 nm, 2.88 ± 0.50 nm, 2.91 ± 0.25 nm, and 2.95 ± 0.25 nm, indicating that all samples exhibit narrow particle size distributions. In terms of temperature dependence, the overall size of N, S-CDs initially increased and then stabilized as the reaction temperature rose. Specifically, at 160 °C, the relatively low reaction temperature resulted in insufficient carbonization, leading to generally smaller carbon dot sizes, while when the temperature was raised to 180 °C, this phenomenon was significantly alleviated, and the particle size increased markedly. Upon further increasing the temperature to 180 °C and above, the sizes of most CDs were concentrated within the 2–3 nm range, with only a small number of particles reaching approximately 4 nm in size, indicating that some degree of particle agglomeration may occur under higher temperature conditions.

**Figure 2:**
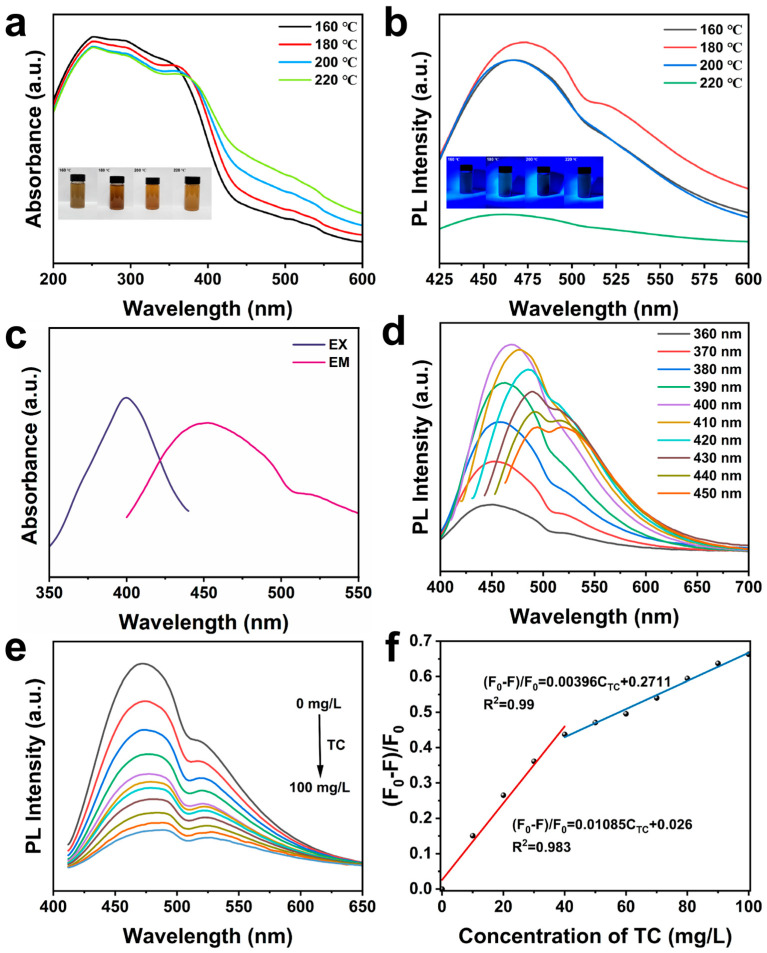
(**a**) UV–Vis absorption spectrum (inset: digital photo of N,S-CDs under visible light) and (**b**) PL spectra of N, S-CDs-X samples at the excitation wavelength of 400 nm (inset: digital photo of N,S-CDs under UV (365 nm) light). (**c**) Fluorescence excitation and emission spectra of N, S-CDs-180. (**d**) Emission spectra of N, S-CDs-180 under different excitation wavelengths. (**e**) Fluorescence spectra of N, S-CDs-180 within 0 to 100 mg/L TC solution. (**f**) Linear relationship between fluorescence value and TC concentration based on the N, S-CDs-180 sample.

The optical properties of N, S-CDs prepared at different reaction temperatures were systematically characterized using photoluminescence (PL) and UV–Vis absorption spectroscopy. Figure 2a exhibits the UV–Vis absorption spectra of liquid N, S-CDs synthesized under different temperature conditions, which indicates the presence of two characteristic absorption peaks in the short-wavelength region (around 250 nm and 300 nm), which can be attributed to the π–π* transition of the C=C bond and the n–π* transition of the C=O/C–N functional groups, respectively [16]. In the long-wavelength region, the N, S-CDs exhibit a distinct broad absorption band accompanied by a kink structure, a feature typically associated with the n–π* transition of the C–S bond, indicating that sulfur has been successfully incorporated into the carbon dot structure [17]. Furthermore, as shown in the inset of Figure 2a, under natural light conditions, as the hydrothermal temperature increased from 160 °C to 220 °C, the color of the N, S-CDs solution gradually shifted from yellow-green to yellow-brown, which is attributed to the increasing degree of carbonization with rising temperature, resulting in the gradual enlargement of the carbon nuclei. In contrast, under 365 nm UV irradiation (inset of Figure 2b), the fluorescence intensity of carbon dots synthesized at different temperatures exhibits a trend of first increasing and then decreasing, with N, S-CDs-180 being the brightest. Combined with the phenomenon described above, this is attributed to the fact that at 160 °C, the low degree of carbonization results in insufficient luminescent centers, while at 220 °C, although the degree of carbonization is high, the reduction in surface functional groups and the increase in defects cause the fluorescence to decrease [18]. As given in Figure 2b, the fluorescence intensity of the synthesized N, S-CDs exhibits a trend of first increasing and then decreasing with rising synthesis temperature, reaching a maximum at 180 °C, which indicates that at this temperature, the structure of the carbon dots (such as the degree of carbonization of the carbon core and the distribution of surface functional groups/defect states) achieves an optimal balance, thereby yielding the best fluorescence performance. Based on a comprehensive evaluation of fluorescence performance, reaction time, and energy consumption, 180 °C was determined to be the optimal synthesis temperature, with a reaction time of 14 h. The N, S-CDs prepared under these optimal conditions was used for subsequent structural characterization and performance testing. Furthermore, as presented in Figure 2c, the maximum excitation wavelength and maximum emission wavelength of this sample were 400 nm and 465 nm, respectively. The optimal excitation wavelength for N, S-CDs is 400 nm, with a maximum emission peak at 465 nm, exhibiting typical wavelength-dependent luminescence behavior. As the excitation wavelength is gradually increased from 360 nm to 450 nm, the fluorescence intensity first increases and then decreases, while the emission peak continuously shifts toward longer wavelengths (red shift), which can be attributed to the synergistic effect of size/structural heterogeneity and differences in surface chemistry caused by N and S co-doping, leading to the selective excitation of different luminescent centers under different excitation conditions. Based on the results of the above fluorescence spectrum tests, the synthesized N, S-CDs-180 was further used to construct a fluorescence (FL) sensing system for the detection of TC. As given in Figure 2e, as the TC concentration increased from 0 to 100 mg/L, a significant fluorescence quenching of N, S-CDs-180 was observed, and the maximum quenching efficiency (*F*_0_ − *F*)/*F*_0_ reached 66.72%, where *F*_0_ and *F* represent the fluorescence intensities at 465 nm before and after the addition of TC, respectively. Furthermore, within the two concentration ranges of 0–40 mg/L and 40–100 mg/L, (*F*_0_ − *F*)/*F*_0_ exhibited an excellent linear relationship with TC concentration (Figure 2f). The corresponding linear fitting equations are (*F*_0_ − *F*)/*F*_0_ = 0.01085*C*_[*TC*]_ + 0.026 (*R*^2^ = 0.983) and (*F*_0_ − *F*)/*F*_0_ = 0.00396*C*_[*TC*]_ + 0.2711 (*R*^2^ = 0.99), with a limit of detection (LOD) of 1.963 mg/L. Based on the low detection limit and wide detection range, the above analytical results demonstrate that N, S-CDs can serve as fluorescent probes for detecting TC.

**Figure 3:**
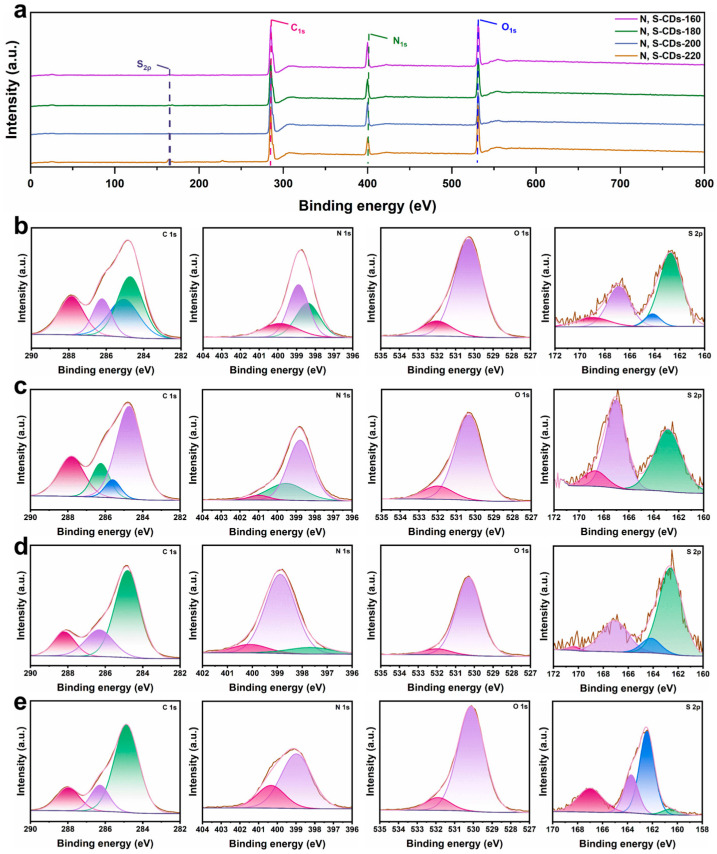
(**a**) XPS survey spectra of N, S-CDs-160, N, S-CDs-180, N, S-CDs-200 and N, S-CDs-220. High resolution spectra of C 1s, N 1s, O 1s and S 2p over (**b**) N, S-CDs-160, (**c**) N, S-CDs-180, (**d**) N, S-CDs-200 and (**e**) N, S-CDs-220.

X-ray photoelectron spectroscopy (XPS) was performed to investigate the elemental composition and surface chemical states of N, S-CDs synthesized at different temperatures. As displayed in Figure 3a, the survey spectra of N, S-CDs-160, N, S-CDs-180, N, S-CDs-200 and N, S-CDs-220 all exhibit the characteristic signals of C 1s, N 1s, O 1s and S 2p, indicating that the obtained CDs are mainly composed of C, N, O and S elements. The presence of N 1s and S 2p signals confirms the successful incorporation of nitrogen and sulfur into the carbon dot system. In addition, the variation in peak intensity among the four samples suggests that the synthesis temperature affects the elemental composition and surface chemical structure of N, S-CDs. The high-resolution XPS spectra of N, S-CDs-160 are given in Figure 3b. The C 1s spectrum can be deconvoluted into several components corresponding to C–C (284.7 eV) [19], C–N (285.6 eV) [20], C–O (286.1 eV) [21] and C=O/C=N (287.8 eV) [22] bonds, indicating the coexistence of a carbonaceous framework and abundant surface functional groups. The N 1s spectrum reveals the presence of different nitrogen configurations, such as pyridinic N (398.4 eV) [23], pyrrolic N (398.9 eV) [24] and graphitic N (399.9 eV) [25], while the O 1s spectrum indicates the existence of oxygen-containing groups including C=O (530.3 eV) [26] and C–O/C–OH (532.0 eV) [27]. In addition, the S 2p spectrum contains sulfur-related species that can be assigned to C–S–C (162.7 eV) [28]/thiophene-like sulfur and oxidized sulfur species such as –SO_x_ (166.9 eV) [29]. However, at the relatively low synthesis temperature of 160 °C, the carbonization and heteroatom doping may be insufficient, which could limit the formation of effective emissive centers. As presented in Figure 3c, N, S-CDs-180 exhibits distinct C 1s, N 1s, O 1s and S 2p components. The C 1s spectrum shows clear contributions from carbon framework structures and heteroatom-related bonds, including C–N/C–S, suggesting that nitrogen and sulfur are effectively incorporated into the carbon dot structure. The N 1s spectrum displays relatively complete nitrogen-related components, indicating the formation of suitable nitrogen-doped structures. These nitrogen species can regulate the electronic structure and introduce emissive surface states. Meanwhile, the O 1s spectrum shows obvious oxygen-containing functional groups, which can improve water dispersibility and passivate surface defects. The S 2p spectrum also shows both C–S-related and oxidized sulfur species, suggesting that sulfur participates in the modulation of the surface chemical environment. The coexistence of appropriate carbonization, N/S co-doping and surface passivation in N, S-CDs-180 is favorable for radiative recombination and is consistent with its strongest fluorescence intensity under UV irradiation. For N, S-CDs-200, the high-resolution spectra are exhibited in Figure 3d. Compared with N, S-CDs-180, the C 1s spectrum becomes more dominated by carbon framework-related components, suggesting an enhanced carbonization degree at higher temperature. The N 1s and S 2p spectra still confirm the existence of nitrogen- and sulfur-containing structures, but the relative distribution of surface chemical states changes with increasing temperature. The O 1s spectrum indicates that oxygen-containing groups are still present, although part of the surface functional groups may undergo dehydration, decarboxylation or structural rearrangement during the higher-temperature hydrothermal process [30], which could weaken the surface passivation effect and disturb the balance of emissive surface states. As shown in Figure 3e, N, S-CDs-220 also contains C, N, O and S-related chemical states, confirming the retention of heteroatom-doped structures at the highest synthesis temperature. Nonetheless, further increasing the temperature may lead to excessive carbonization and more pronounced rearrangement or loss of surface functional groups. The changes in C 1s, N 1s, O 1s and S 2p components suggest that the surface chemical environment of N, S-CDs-220 differs from that of N, S-CDs-180. Excessive carbonization can reduce the number of suitable emissive surface states and enhance non-radiative recombination pathways, leading to a decrease in fluorescence intensity. Overall, the XPS results indicate that the synthesis temperature plays an important role in regulating the carbonization degree, heteroatom doping state and surface functional groups of N, S-CDs. Among the four samples, N, S-CDs-180 shows a more favorable balance between carbon core formation, N/S co-doping and surface passivation, which explains its superior fluorescence performance compared with N, S-CDs-160, N, S-CDs-200 and N, S-CDs-220.

**Figure 4:**
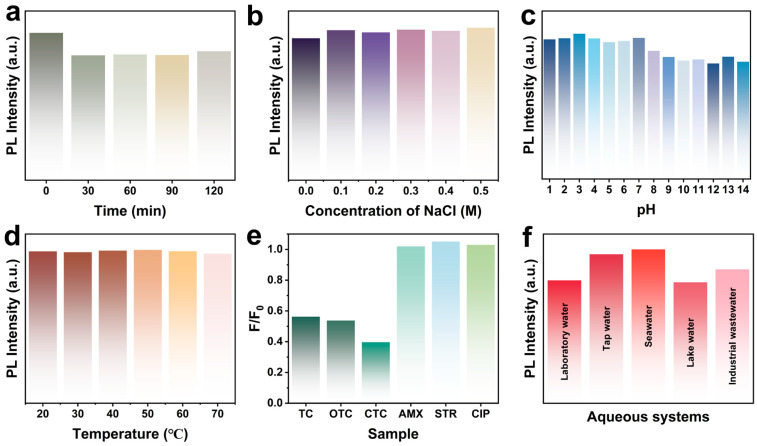
(**a**) Fluorescence intensity of N,S-CDs-180 versus time. (**b**) Fluorescence intensity of N,S-CDs-180 in NaCl solution with different concentrations. (**c**) Fluorescence intensity of N,S-CDs-180 at different pH values. (**d**) Fluorescence intensity of N,S-CDs-180 at different temperatures. (**e**) Selectivity of N, S-CDs-180 as the fluorescent probe for fluorescence detection of antibiotics. (**f**) Extinction coefficients of N, S-CDs-180 for detecting TC in different aqueous systems.

To investigate the stability of N, S-CDs-180 under various conditions, their photostability, salt tolerance, pH tolerance, and thermal stability were evaluated. Specifically, N, S-CDs-180 were exposed to continuous irradiation at 365 nm (Xenon lamp equipped with a 365 nm filter), and the fluorescence intensity was recorded every 30 min. As presented in Figure 4a, the fluorescence intensity changed only slightly over the irradiation period, indicating that N, S-CDs-180 possess outstanding photostability. Subsequently, the fluorescence intensity of N, S-CDs-180 was measured in saline solutions with different ionic strengths (Figure 4b). Only minimal fluctuations were observed, demonstrating that N, S-CDs-180 exhibit strong tolerance to high ionic strength. The effect of pH on the fluorescence intensity of N, S-CDs-180 was further investigated (Figure 4c), and the fluorescence intensity remained high and stable when the pH ranged from 6 to 8, indicating that near-neutral conditions are more favorable for the photoluminescence of N, S-CDs-180. Therefore, unless otherwise stated, all fluorescence measurements were performed at pH = 7. In addition, the effect of temperature on the photoluminescence of N, S-CDs-180 was examined in Figure 4d, and the fluorescence intensity remained essentially constant between 20 °C and 70 °C, suggesting excellent thermal stability within this range. Accordingly, the characterization and subsequent measurements of N, S-CDs-180 were conducted at 25 °C and pH = 7. In addition, to verify the specificity of N, S-CDs-180, the fluorescence response of N, S-CDs was tested using oxytetracycline (OTC), chlortetracycline (CTC), amoxicillin (AMX), streptomycin (STR), and ciprofloxacin (CIP) as control samples. As displayed in Figure 4e, under identical conditions, the fluorescence intensity of N, S-CDs-180 decreased to 0.56, 0.54 and 0.40, respectively, upon the addition of TC, OTC, and CTC, indicating that these three tetracycline antibiotics effectively quenched the fluorescence of N, S-CDs-180. In contrast, AMX, STR and CIP caused almost no change in fluorescence intensity, with *F*_0_/*F* values all approaching 1. Based on this, N, S-CDs-180 demonstrates excellent targeted recognition capability and high sensitivity toward tetracycline antibiotics, rendering them suitable for the specific detection of such compounds. To further evaluate the fluorescence stability of N, S-CDs-180 in real aquatic environments, the material was dispersed in several common aqueous solutions (Figure 4f), and the corresponding fluorescence intensities were measured. The results reveal that the fluorescence intensity of N, S-CDs-180 remains largely unchanged across different aqueous systems, with only minimal variation among samples. Notably, even in complex industrial wastewater, the material retains strong fluorescence intensity. Consequently, the as-prepared N, S-CDs exhibit outstanding environmental adaptability, and their stable fluorescence signal provides a reliable basis for the detection of tetracycline antibiotics in diverse aquatic environments.

**Figure 5:**
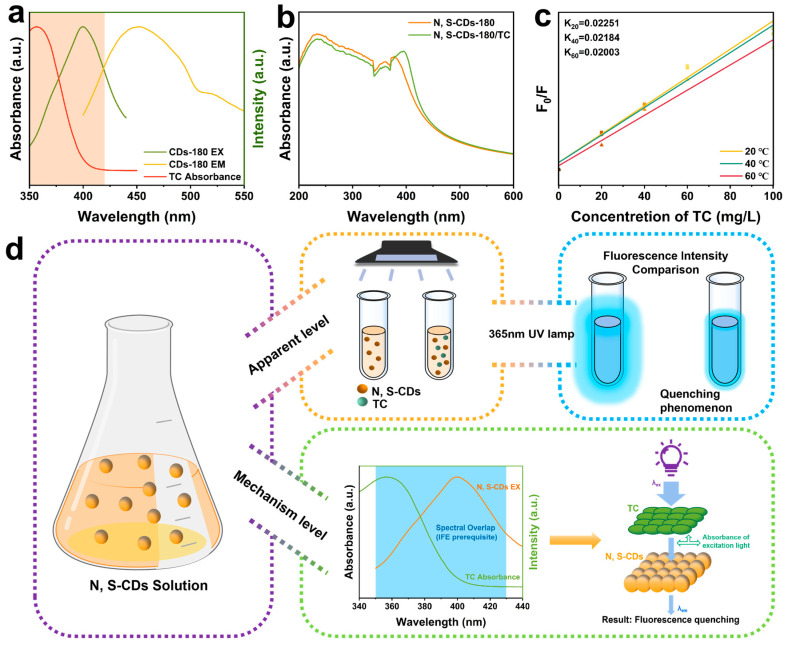
(**a**) Spectral overlap region of TC absorption (UV–Vis) and N, S-CDs-180 fluorescence (excitation/emission). (**b**) UV–Vis absorption spectra of N, S-CDs blended with TC. (**c**) Stern–Volmer curves of N, S-CDs-180 quenched by TC at 20, 40 and 60 °C. (**d**) Schematic illustration of the mechanism for TC detection by N, S-CDs fluorescence.

To further investigate the sensing mechanism of N, S-CDs/TC system, a series of characterization analyses and theoretical calculations were conducted. First, the UV absorption spectrum of TC was measured and compared it with the normalized excitation and emission spectra of N, S-CDs-180 (Figure 5a), which revealed that the excitation spectrum of N, S-CDs-180 overlaps with the UV–Vis absorption spectrum of TC in the 350–420 nm range (shaded area in the figure), indicating that the blue quenching of N, S-CDs may be related to the inner filter effect (IFE) of TC on N, S-CDs. Meanwhile, after performing UV–Vis absorption measurements on the N, S-CDs-180-TC system and comparing them with the UV–Vis spectrum of N, S-CDs-180 (Figure 5b), no new absorption peaks were observed, which indicates that no new chemical bonds were formed between N, S-CDs-180 and TC, ruling out the possibility of static quenching and further confirms the previous IFE hypothesis. Furthermore, the changes in fluorescence intensity of the N, S-CDs-180-TC system at 20, 40 and 60 °C were measured, plotting the Stern–Volmer curves as shown in Figure 5c. The slopes were *K*_20_ = 0.02251, *K*_40_ = 0.02184, and *K*_60_ = 0.02003, respectively, indicating that they remained essentially unchanged, which confirms that IFE plays a dominant role in this system [31]. Based on the experimental results presented above, Figure 5d illustrates the visual fluorescence comparison and the underlying quenching mechanism. At the microscopic level, the excitation spectrum of the N, S-CDs overlaps significantly with the absorption spectrum of TC. Consequently, when the system is irradiated with excitation light, TC competes with the N, S-CDs for photon absorption, effectively acting as a primary filter that attenuates the light intensity available to excite the carbon dots. This reduction in effective excitation leads to a decreased fluorescence intensity detected by the instrument, thereby manifesting as apparent fluorescence quenching at the macroscopic level.

## 3. Experimental Section

### 3.1. Materials and Reagents

The shuttlecocks were obtained from the gymnasium at Jiangsu University of Science and Technology. Tetracycline (99%), oxytetracycline (99%), chlortetracycline (99%), amoxicillin (99%), streptomycin (99%), ciprofloxacin (99%) and ethanol were all purchased from Aladdin Industrial Co., Ltd. (Shanghai, China).

### 3.2. Synthesis of N, S-CDs

Poultry feathers recovered from shuttlecocks discarded at the badminton court of Jiangsu University of Science and Technology were used as raw materials. N, S-CDs were synthesized via a facile hydrothermal method based on the previous report from our group [12]. The steps are as follows. Before use, the collected feathers were manually cleaned to remove visible impurities and then washed in 35% ethanol solution under magnetic stirring for 5 min to reduce the influence of surface dust, grease, and possible residual processing agents. Subsequently, the feathers were rinsed with deionized water, dried, and cut into small pieces before the hydrothermal reaction. First, 0.28 g of feathers and 30 mL of deionized water were mixed and added to an autoclave; secondly, after being placed in an oven, it was hydrothermally treated at 160 °C, 180 °C, 200 °C and 220 °C for 14 h to obtain the N, S-CDs solution and then cooled to room temperature. Finally, for removing the residue, the obtained N, S-CDs was filtered and placed in a freeze dryer and freeze-dried at −50 °C for 24 h to produce N, S-CDs solid powder (denoted as N, S-CDs-160, N, S-CDs-180, N, S-CDs-200 and N, S-CDs-220).

### 3.3. Fluorescence Detection

To investigate the effect of TC concentration on the fluorescence intensity of N, S-CDs, 100 mL of N, S-CDs solution (5 mg/mL) was prepared for fluorescence measurements. TC was then added to the N, S-CDs solution at varying concentrations in laboratory deionized water, lake water and seawater to simulate different aqueous environments, and the corresponding fluorescence spectra were recorded. The fluorescence quenching behavior of N, S-CDs as a function of TC concentration was analyzed by a fluorescence detector (F98-fluorospectrophotometer), and the linear relationship between TC concentration and the fluorescence response (F/F_0_) was further evaluated.

### 3.4. Characterization Methods

Transmission electron microscopy (TEM) characterization was performed using an FEI-TecnaiTM G2F30 system from Hillsboro, OR, USA equipped with a field emission gun, operating at a voltage of 200 kV. X-ray photoelectron spectroscopy (XPS) measurements were performed using a VG Multilab 2000 instrument from East Grinstead, England equipped with an Al Ka source to determine the chemical composition. The measured ultraviolet–visible (UV–Vis) spectra were recorded using a Shimadzu UV-3100 spectrophotometer from Kyoto, Japan to assess the optical properties of the materials in the wavelength range of 200 to 600 nm.

## 4. Conclusions

In summary, we reported a bio-based N, S-CDs synthesized via a facile one-step hydrothermal method without the need for additional reactants or catalysts. Comprehensive characterizations revealed that the properties of the N, S-CDs are governed by the synergistic interplay between surface functional groups and the degree of carbonization, which varies with the hydrothermal temperature. Consequently, the N, S-CDs synthesized at 180 °C (N, S-CDs-180) were found to exhibit an excellent linear fluorescence quenching response to TC, along with superior stability and selectivity. This work provides a meaningful strategy for designing highly sensitive and cost-effective fluorescent probes, holding great significance for the quantitative detection of TC antibiotics.

## Data Availability

The raw data supporting the conclusions of this article will be made available by the authors on request.
